# Weaning from NIV: how rapidly can we go?

**DOI:** 10.1186/cc9569

**Published:** 2011-03-11

**Authors:** J Chico, L Sayagues, R Casado, M Muñoz, L Lage, S Vara, V Gomez, C Vara

**Affiliations:** 1 Hospital Xeral, Vigo, Spain; 2 Hospital Clinico, Santiago Compostela, Spain

## Introduction

Little evidence exists about how to wean patients from NIV. We assess the efficacy and tolerance of a rapid weaning sequence.

## Methods

The population was consecutive patients admitted to our ICU during 1 year with COPD or pulmonary edema (PE) who underwent NIV. Criteria for weaning: improvement of acute disease, pH >7.33, RR <25, pO_2 _> 65, FiO_2 _< 0.6, EPAP <8. Day 1: withdrawal of NIV (could be placed for a maximum of 8 hours the first day). If no reintroduction of NIV: discharge. If reintroduction <8 hours: observation for 24 hours more, and discharge if no need for NIV. If deterioration or need to extend NIV time: change to a standard protocol (decremental NIV time).

## Results

Twenty-one patients were included, 89% male. Mean age was 67 years. Fifty per cent had previous history of COPD, 25% heart disease (mostly ischemic and hypertensive) and 35% both. Reason for admission was PE (80%), and 20% COPD exacerbation. Mean APACHE II score: 20. Mean FiO_2_/pH/pCO_2_: 0.6/7.22/72. Mean EPAP/IPAP: 6/19. Mean time of NIV: 48 hours. Mean time of weaning: 35 hours. Eighteen patients were weaned successfully in 48 hours (50% discharged in 24 hours). No patient needed readmission. We found no differences in weaning success related with NYHA, APACHE, reason for admission or NIV time. All patients with PE were weaned successfully. Mean basal LVEF: 54%. Mean LVEF in acute disease: 50%. Those with LVEF deterioration showed the same success rates. Patients with history of severe COPD (FVC <38%) needed more NIV time during weaning, longer ICU stay and were more likely to fail weaning (three patients failed weaning, all with severe COPD).

## Conclusions

Evidence about how to wean patients from NIV is scarce. The usual practice is to decrease NIV time, but extending weaning time can lead to higher costs and NIV failure. Pinto showed in 65 COPD patients that a 3-day approach with decreasing time of NIV was feasible. All patients were discharged in 4 days without complications. In our case, a more aggressive approach was attempted. Our results suggest that rapid weaning sequence could be feasible in PE patients, although further studies are needed.

